# Polymicrobial Arthritis Following a Domestic Cat Bite Involving *Rahnella aquatilis* in an Immunocompetent Patient

**DOI:** 10.3390/microorganisms13081725

**Published:** 2025-07-23

**Authors:** Olivier Nicod, Marie Tré-Hardy, Bruno Baillon, Ingrid Beukinga, William Ngatchou, Nada Riahi, Laurent Blairon

**Affiliations:** 1 Department of Orthopedics, Iris Hospitals South, 1050 Brussels, Belgium; 2 Université libre de Bruxelles, 1050 Brussels, Belgium; 3 Department of Pharmacy, Namur Research Institute for Life Sciences, University of Namur, 5000 Namur, Belgium; 4 Department of Emergency Medicine, Iris Hospitals South, 1050 Brussels, Belgium; 5 Department of Laboratory Medicine, Iris Hospitals South, 1050 Brussels, Belgium

**Keywords:** cat bite, join infection, *Erwiniaceae*, feline oral flora, metagenomics, antibiotic resistance, microbial diversity

## Abstract

Cat bites frequently lead to polymicrobial infections due to deep puncture wounds that inoculate oral flora into poorly oxygenated tissues. While *Pasteurella multocida* is the most commonly implicated organism, environmental and atypical pathogens may also play a role, yet often go unrecognized. This article reports a rare case of polymicrobial septic arthritis caused by a domestic cat bite in an immunocompetent adult, with isolation of *Rahnella aquatilis*, a freshwater-associated *Enterobacterales* species not previously reported in this context. A 33-year-old immunocompetent male presented with acute hand swelling, pain, and functional impairment within 24 h of the bite. Emergency surgery revealed purulent tenosynovitis and arthritis. Intraoperative cultures identified *R. aquatilis*, *P. multocida*, and *Pantoea agglomerans*. Identification was performed using MALDI-TOF MS. The *R. aquatilis* isolate was susceptible to beta-lactams (excluding ampicillin), quinolones, and co-trimoxazole. The patient received amoxicillin–clavulanic acid and fully recovered within two weeks. This is the first reported case of joint infection involving *R. aquatilis* following a cat bite. It highlights the importance of considering environmental *Enterobacterales* in animal bite wounds, and the utility of advanced microbiological tools for detecting uncommon pathogens. Broader awareness may improve diagnosis and guide targeted therapy in polymicrobial infections.

## 1. Introduction

Animal bites are a common cause of emergency department visits and may result in infections of varying severity. Although dog bites are more frequent, cat bites have a particularly high risk of infection. This elevated risk is due to the unique morphology of feline dentition, which causes narrow, deep puncture wounds that can inoculate bacteria into poorly oxygenated spaces such as tendon sheaths and joint capsules [1].

Infections following cat bites are typically polymicrobial. The most frequently isolated pathogen is *Pasteurella multocida*, followed by *Streptococcus*, *Staphylococcus*, *Moraxella*, and several anaerobes [2,3,4,5]. These organisms originate primarily from the cat’s oral flora. While the clinical presentation and microbiological features of such infections are well described, most of the literature focuses on commonly isolated bacteria, and the involvement of less conventional organisms remains largely underdocumented.

Some authors have reported secondary infections of varying severity following cat bites caused by much rarer bacteria such as *Bacteroides pyogenes*, *Bergeyella species*, and NO-1 [6,7,8,9,10]. In addition, recent investigations of the feline oral microbiota using 16S rRNA gene sequencing have significantly expanded the spectrum of known bacterial species colonizing the mouths of both healthy cats and those with chronic gingivostomatitis. These studies have revealed previously uncharacterized phylotypes and novel bacterial taxa not detectable by conventional culture methods [11]. The ability to identify such organisms highlights the increasing microbial diversity associated with the feline oral cavity and, consequently, broadens the range of potential pathogens that may be transmitted through animal bites.

*Rahnella aquatilis* is a Gram-negative, facultatively anaerobic rod belonging to the order *Enterobacterales*. Originally isolated from freshwater sources, this organism is rarely reported as a human pathogen. When implicated, it has been associated with infections such as bacteremia, urinary tract infections, and surgical wound infections, primarily in immunocompromised individuals. To date, there have been no reports of *R. aquatilis* in the context of animal bite-related arthritis.

The aim of this case report is to emphasize the importance of not overlooking co-infecting organisms in animal bite wounds, including environmental bacteria that may transiently colonize the animal’s oral cavity and subsequently infect humans. By highlighting such atypical pathogens, we aim to raise awareness of their clinical relevance and the need for broad-spectrum diagnostic and therapeutic approaches in managing polymicrobial infections.

## 2. Case Presentation

### 2.1. Clinical Presentation

A 33-year-old self-employed male presented for urgent orthopedic consultation following cat bites sustained the previous day on the palmar and dorsal aspects of the proximal interphalangeal (PIP) joints of the second and fifth fingers of the left hand. The patient was immunocompetent with no relevant medical history, except for a spontaneous pneumothorax one year prior, after which he had ceased smoking tobacco and using cannabis.

He was referred by his primary care physician for rapidly progressing symptoms, including significant swelling, erythema, and severe pain exacerbated by passive extension of the affected fingers. These symptoms were more pronounced in the fifth finger, with pain radiating to the PIP joints of both the second and fifth fingers. Percussion tenderness extended proximally toward the carpal tunnel.

### 2.2. Surgical Findings

**Figure 1:**
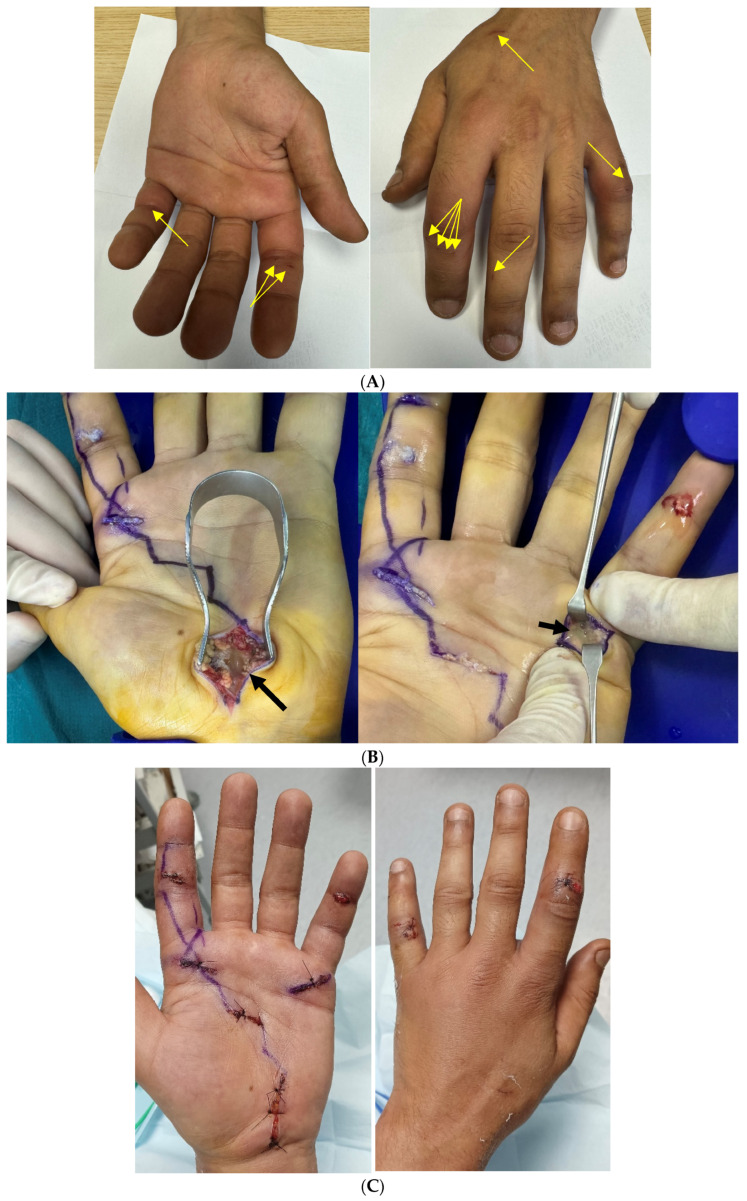
(**A**) Preoperative period. Palmar aspect: No visible abnormalities except for localized erythema over the proximal phalanx of the little finger. Dorsal aspect: A few bite marks are observed on the index and middle fingers. While no apparent abnormalities are visible, clinical findings strongly suggest significant underlying damage. Yellow arrows point to the locations of the bite wounds. (**B**) Intraoperative findings. Carpal tunnel was incised due to severe percussion-induced pain, revealing purulent fluid (large arrow). The flexor tendon sheath of the little finger was opened at the level of the A1 pulley, with purulent fluid clearly visible between the retractors (small arrow). Blue lines indicate possible incision pathways as per Brüner’s zigzag approach. (**C**) Postoperative, day 2. Wound closure was achieved using loose sutures.

In an outpatient setting, emergency surgical drainage was performed in the operating theater under locoregional anesthesia. Each bite wound was debrided, revealing purulent fluid at all affected sites (Figure 1).

A PIP joint arthrotomy of the fifth finger also expressed turbid synovial fluid. On the palmar aspect of the involved fingers, wound exploration revealed purulent fluid tracking along the flexor tendon sheaths.

A decision was made to surgically release the carpal tunnel, which also revealed purulent fluid. Signs of infection were evident along the entire length of the flexor tendon sheaths, extending from proximal to distal.

Multiple samples were collected from each site for microbiological analysis, followed by thorough saline irrigation and loose wound closure using non-absorbable sutures. The same day, the patient was discharged with oral broad-spectrum antibiotic monotherapy (amoxicillin–clavulanic acid 875 mg three times daily for 6 weeks) and analgesics.

### 2.3. Microbiological Analysis

**Figure 2:**
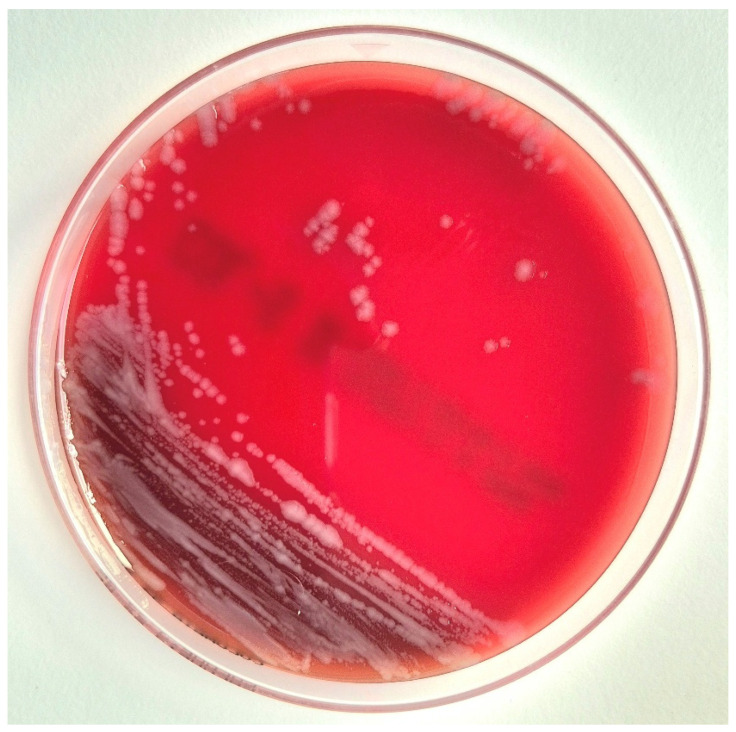
Growth of *R. aquatilis* on Columbia blood agar after 24 h of incubation at 37 °C. Colonies appear smooth, grayish, and non-hemolytic.

Bacterial cultures identified *P. multocida* in purulent samples from the index finger and carpal tunnel. Samples from the fifth finger yielded both *P. multocida* and *Pantoea agglomerans*. Additionally, pus from the PIP joint of the fifth finger grew *R. aquatilis*, identified by matrix-assisted laser desorption/ionization time-of-flight mass spectrometry (MALDI-TOF MS) (score 2.2). The appearance of *R. aquatilis* colonies is illustrated in Figure 2.

According to EUCAST 2022 interpretation, this strain was resistant to ampicillin but susceptible to amoxicillin–clavulanic acid, broad-spectrum beta-lactams (piperacillin–tazobactam, third- and fourth-generation cephalosporins, carbapenems, monobactam), quinolones, aminoglycosides, and co-trimoxazole. Regarding the other bacteria found in the samples, *P. multocida*—of which only a few colonies grew in culture—was susceptible to penicillin, amoxicillin–clavulanic acid, tetracyclines, and co-trimoxazole. *P. agglomerans* exhibited a wild-type susceptibility profile, identical to that of *Rahnella*.

### 2.4. Treatment and Outcome

The postoperative course was uneventful, with appropriate antibiotic therapy maintained throughout. The patient received standard wound care at home three times a week and had weekly orthopedic follow-ups with his surgeon. To prevent postoperative stiffness and swelling, immediate and complete hand mobilization was encouraged.

At the first postoperative visit two days after surgery, the patient had regained full hand motion. Physiotherapy was proposed, but the patient was eager to return to his manual work. He resumed work after suture removal at two weeks, with a full range of motion and healed wounds.

## 3. Discussion

Animal bites are a common cause of emergency department visits, accounting for 1% of cases annually in the USA, with an estimated cost exceeding USD 50 million. Most bites are caused by dogs, while cat bites represent only 5% to 10% of cases [2]. Cat bites, however, are more likely to become infected, with reported infection rates ranging from 28% to 80%, depending on the study. These infections typically occur within 24 h after a dog bite and within 12 h after a cat bite. They may be localized (subcutaneous abscess, tendinitis, septic arthritis, osteomyelitis) or, more rarely, systemic, potentially leading to severe complications such as sepsis, endocarditis, meningitis, or brain abscess. The hand is affected in 50% of dog bites and 63% of cat bites [3].

Although cat bites are less destructive than dog bites due to their weaker jaw strength, their sharp, penetrating teeth frequently introduce oral flora deep into poorly oxygenated tissues, leading to polymicrobial infections and increasing the risk of complications such as septic arthritis and osteomyelitis [1]. As a result, the organisms involved most often originate from the biter’s oral flora, and more rarely from the victim’s skin flora.

In our case report, multiple organisms were isolated from the wounds, including *P. multocida*, as expected in cat bite infections, as well as *P. agglomerans* and *R. aquatilis*. *P. agglomerans* (formerly *Enterobacter agglomerans*) is an environmental bacterium within the family *Erwiniaceae*, within the same family as *Rahnella*, that is rarely implicated in wound infections and, to the best of our knowledge, has not been associated with animal bites [12]. A recent study of the feline oral microbiome, based on swabs collected from the oral cavities of 11 cats, identified not only traditional pathogens such as *Pasteurella*, *Moraxella*, and *Neisseria*, but also a wide range of bacterial species from various other phyla. In total, 273 genera belonging to 18 bacterial phyla were detected. Eight phyla accounted for 97.6% of all sequences: *Proteobacteria* (75.2%), *Bacteroidetes* (9.3%), *Firmicutes* (6.7%), SR1 (2.7%), *Spirochaetes* (1.8%), *Fusobacteria* (1.3%), and *Actinobacteria* (0.6%). *Proteobacteria* comprise a wide range of families, including *Enterobacteriaceae*. Although *R. aquatilis* was not identified in this particular study, its presence in the feline oral microbiome cannot be entirely ruled out [13]. To date, the genus *Rahnella* has not been isolated from infected bite wounds. *R. aquatilis* is a facultative Gram-negative rod within the order *Enterobacterales.* First described in 1976 by Gavini et al. and named after bacteriologist Otto Rahn, it is primarily aquatic in origin [12]. Human infections due to *Rahnella* are rare and generally involve bacteremia, with occasional reports of endocarditis, and gastrointestinal, urinary, or surgical wound infections. Such cases predominantly affect immunocompromised patients or those with underlying comorbidities [14,15,16]. Severe infections in immunocompetent individuals are rare and have been linked to the injection of contaminated fluids or parenteral nutrition [17,18,19]. The detection of *R. aquatilis* in this case raises important questions regarding the ecological niche and pathogenic potential of this environmental bacterium. Traditionally associated with freshwater habitats, *R. aquatilis* is virtually absent from standard lists of zoonotic or bite-related pathogens. Its isolation from purulent synovial fluid in a sterile joint environment suggests a genuine role in the infectious process rather than incidental colonization. The ability to identify *R. aquatilis* with high confidence in this context was made possible by the use of MALDI-TOF MS, which has significantly expanded the scope of species-level microbial identification in clinical microbiology. Prior to the routine use of MALDI-TOF, rare or atypical *Enterobacterales* such as *Rahnella* were often misclassified as part of the *Enterobacter cloacae* complex or overlooked entirely. As such, it is plausible that *R. aquatilis* and similar species have been historically underreported in bite-related infections, not due to true absence, but due to limitations in diagnostic resolution. The increasing use of MALDI-TOF MS, and potentially next-generation sequencing, may reveal a broader spectrum of zoonotic bacteria involved in polymicrobial wound infections than previously appreciated. Our case also illustrates the polymicrobial synergy frequently observed in bite-related infections. While *R. aquatilis* is not known for its virulence in humans, it may have contributed to the inflammatory process by amplifying tissue invasion or delaying immune clearance in the presence of more established pathogens like *P. multocida*. However, genomic and proteomic studies conducted in aquatic animals have identified *R. aquatilis* clones carrying multiple resistance and virulence genes, suggesting a pathogenic potential that may be underrecognized in human infections [20]. Our patient was a healthy young adult with no significant medical history other than a prior spontaneous pneumothorax. As a self-employed manual laborer, an early return to full hand function was essential for resuming his professional activities. The clinical course was notable for the rapid progression of symptoms (severe pain, swelling, and functional impairment) within less than 24 h after the injury. The four Kanavel signs—(1) the digit held in slight flexion at rest, (2) fusiform swelling, (3) tenderness along the flexor tendon sheath, and (4) pain with passive extension—are traditionally used to identify pyogenic flexor tenosynovitis. In our patient, three of these signs were present: the finger was maintained in a flexed posture, there was marked tenderness over the sheath, and passive extension elicited significant pain. However, fusiform swelling was absent because the patient presented very shortly after the bite occurred. Although the absence of one Kanavel sign may reduce the clinical suspicion, it does not exclude the diagnosis, as not all patients exhibit all four signs, particularly in early-stage presentations [21]. Notably, the patient had not received antibiotic prophylaxis before referral. Regarding antibiotic management, current guidelines recommend amoxicillin–clavulanate for both prophylaxis and treatment of animal bite wounds [2,22,23]. While the prophylactic use of antibiotics has shown mixed results overall, several studies support its efficacy specifically for hand injuries, which carry a higher risk of infection [22]. Treatment failures involving atypical Gram-negative organisms remain rarely reported in the literature. In our case, the susceptibility profile of *R. aquatilis* and co-isolated pathogens to amoxicillin–clavulanate supported the decision to continue this treatment, which resulted in complete clinical recovery without complications. It is important to note that the patient received prompt medical management following the bite, which is a known prognostic factor for favorable outcomes. Delayed intervention may lead to more complex surgical procedures and, in some cases, even amputation, particularly in diabetic patients with peripheral neuropathy [24]. In addition to surgical drainage, which plays a key role in the management of an infected hand bite wound, some authors recommend the use of corticosteroids to reduce swelling [23]. Temporary immobilization of the hand is sometimes advised, and physiotherapy plays an important role in the postoperative period.

Our study has several limitations. Oral swabs from the cat’s oral cavity would have enabled confirmation of the presence of the implicated species in the animal’s oral flora and supported its role in the infection. However, given that *Rahnella aquatilis* is an environmental organism, it is also possible that its presence represented only transient colonization. As such, a culture of the cat’s oral flora performed days after the bite might not have revealed the bacterium, even if it had been present at the time of the incident. Further investigations are needed to comprehensively characterize the microbiota of the feline oral cavity, including species-level sequencing and the analysis of microbial interactions. Currently, only cultivable species on agar media are identified in clinical laboratories, which likely underrepresent the actual microbial diversity. The discovery and sequencing of the human oral microbiome are relatively recent, and they have significantly enhanced our understanding of the pathogenic potential of species that exist not in isolation but in complex communities [25]. Similar approaches applied to the feline oral microbiome could provide insights into pathogenic mechanisms. Finally, the identification of virulence genes in *R. aquatilis* is essential to better understand its pathogenicity. However, as with the detection of antimicrobial resistance genes, such investigation requires next-generation sequencing (NGS) and specialized laboratory infrastructure, which are not currently available in routine clinical settings. Moreover, this report is based on a single patient, and no similar cases have been documented to date. Further case reports and clinical observations involving animal bite-related infections would be valuable in determining whether *R. aquatilis* should be regarded as a clinically relevant pathogen in this context.

## 4. Conclusions

This report describes the first documented case of arthritis that may involve *Rahnella* following a domestic animal bite. Clinicians should consider atypical Gram-negative bacteria as possible culprits in rapidly evolving bite-related infections, even in otherwise healthy individuals.

The patient made a full recovery following a comprehensive management strategy that combined early surgical intervention with targeted antibiotic therapy (amoxicillin–clavulanate), guided by microbiological documentation.

Advances in metagenomic techniques, particularly NGS and shotgun metagenomics, offer new opportunities to explore the full microbial spectrum associated with bite-related infections. These approaches allow for the detection of uncultivable or previously uncharacterized organisms that may coexist with known pathogens. However, their implementation in routine clinical microbiology remains limited due to high instrumentation costs, technical demands, and the need for advanced bioinformatics infrastructure and expertise.

## Data Availability

The original contributions presented in this study are included in the article. Further inquiries can be directed to the corresponding author.
